# Circulating endothelial cells in oncology: pitfalls and promises

**DOI:** 10.1038/sj.bjc.6604383

**Published:** 2008-05-27

**Authors:** M H Strijbos, J W Gratama, J Kraan, C H Lamers, MA den Bakker, S Sleijfer

**Affiliations:** 1 Department of Medical Oncology, Erasmus University Medical Center, Rotterdam, The Netherlands; 2 Department of Pathology, Erasmus University Medical Center, Rotterdam, The Netherlands

**Keywords:** CEC, endothelium; angiogenesis, vasculature, biomarker, endothelial cells

## Abstract

Adequate blood supply is a prerequisite in the pathogenesis of solid malignancies. As a result, depriving a tumour from its oxygen and nutrients, either by preventing the formation of new vessels, or by disrupting vessels already present in the tumour, appears to be an effective treatment modality in oncology. Given the mechanism by which these agents exert their anti-tumour activity together with the crucial role of tumour vasculature in the pathogenesis of tumours, there is a great need for markers properly reflecting its impact. Circulating endothelial cells (CEC), which are thought to derive from damaged vasculature, may be such a marker. Appropriate enumeration of these cells appears to be a technical challenge. Nevertheless, first studies using validated CEC assays have shown that CEC numbers in patients with advanced malignancies are elevated compared to healthy controls making CEC a potential tool for among other establishing prognosis and therapy-induced effects. In this review, we will address the possible clinical applications of CEC detection in oncology, as well as the pitfalls encountered in this process.

Angiogenesis is thought to be an absolute prerequisite for the growth and dissemination of malignant tumours. When a tumour reaches a size of 1–2 mm^3^, its microenvironment can no longer provide the required amount of nutrients and oxygen by diffusion. The resulting cellular hypoxia initiates a response in malignant cells: the so-called ‘angiogenic switch’ (Naumov *et al*, 2006). By upregulating the expression of proangiogenic proteins, the tumour induces sprouting of pre-existing capillaries, which result in the formation of new vessels.

The pivotal role of angiogenesis in tumour growth prompted the development of several agents targeting receptors or signal transduction pathways involved in angiogenesis. Based on data from randomised clinical trials, the benefit of compounds that inhibit angiogenesis has already been demonstrated in several types of cancer, including metastatic colon carcinoma (Kabbinavar *et al*, 2003), renal cell carcinoma (Motzer *et al*, 2007), and non-small lung cell carcinoma (Sandler *et al*, 2006). In addition to these drugs inhibiting angiogenesis, another class of anticancer drugs, vascular disrupting agents (VDAs), has recently been developed and is designed to target already established tumour vasculature. Clinical studies exploring the latter drugs are currently ongoing.

Given the important role of angiogenesis in oncology in terms of pathogenesis as well as being a target for treatment, there is an increasing need for markers that accurately reflect effects impacting tumour vasculature. A promising candidate to serve as such a marker is the enumeration of circulating endothelial cells (CEC). CEC are mature endothelial cells, sloughed off the vessel wall as a result of vascular insults. Their number in peripheral blood is considered to reflect the extent of vascular damage in patients with vasculopathies. Recently, several assays for their detection and quantification have been developed. Although consensus on the phenotypic definition of CEC as well as on the optimal enumeration technique is still lacking, the number of clinical studies assessing CEC in cancer patients is rapidly expanding. Several studies on CEC also incorporate a strategy to enumerate endothelial progenitor cells (EPC), which, in contrast to CEC, are thought to originate from the bone marrow and to contribute actively to angiogenesis. A study by Lyden showed in a murine model that, all tumour vessel endothelial cells were bone marrow-derived (Lyden *et al*, 2001), and similar observations were done in humans (Peters *et al*, 2005), stressing the importance of EPC in angiogenesis. However, as the frequency of EPC in blood is suggested to be several times lower than those of CEC, while additionally, their exact phenotype has not been elucidated, it is very difficult to reliably detect and enumerate EPC by currently available assays. EPC are therefore not included in this article.

This review provides an overview of the uses of CEC as surrogate marker for vascular damage in oncology, in particular technical issues concerning their detection, its potential applications, and results from clinical studies obtained so far.

## TECHNICAL ISSUES IN CEC ENUMERATION

Currently, there are several assays described for the detection and enumeration of CEC. All these assays have to deal with the low number of CEC in peripheral blood, (typically 0–20 CEC per ml in healthy donors) rendering such assays highly susceptible to errors in sampling, preparation, and analysis. For instance, several groups have demonstrated the negative impact of venipuncture, as traumatically detached CEC contribute significantly to CEC counts (Goon *et al*, 2006; Rowand *et al*, 2007). Because of this low number, enrichment steps are applied in several approaches, which inevitably leads to cell loss and underestimation of the actual CEC number. Next to proper sampling, thorough analysis of enrichment efficacy, reported in terms of purity and recovery is therefore mandatory before using such assays based on enrichment in the clinic. The EUROCEC network has provided useful suggestions on how such a validation might be performed in practise (www.eurocec.com)(Woywodt *et al*, 2006).

Of key importance for proper detection and enumeration of CEC is the use of specific markers. However, the endothelium is a highly dynamic structure, closely involved in haemostasis, inflammation, regulation of vascular tonus, and angiogenesis. This dynamic process is accompanied by changing immunophenotype of endothelial cells that may largely account for the large number of different CEC phenotypes reported in literature. For enumeration of the total CEC number, and not a particular subpopulation, it is necessary to identify markers that are specific for and are constantly expressed by all CEC. Currently, no marker is considered to meet these criteria. Consequently, assays rely on multiple characteristics to define CEC. The majority of current assays define CEC as being positive for the CD146 antigen, present on endothelial cells, but also on a subset of activated T-cells, and melanomas (Elshal *et al*, 2005). Table 1 provides an overview of markers frequently used in CEC assays. As the different reported phenotypes render it impossible to compare results between various studies, consensus on a common endothelial cell phenotype is a key issue.

Another mandatory step in the development of CEC assays is the validation of the true endothelial origin of cells designated CEC by that assay. For this purpose, several unique features of endothelial cells can be used. These include uptake of Ulex Europaeus Lectin-1, or UEA-1 (Goon *et al*, 2006) or the ability of CEC to scavenge acetylated low-density lipoproteins, which can be visualised when labelled with 1,1′-dioctadecyl-3,3,3′,3′-tetramethyl-indocarbocyanine perchlorate (Dil Ac-LDL) (Voyta *et al*, 1984). In addition, immunohistochemistry or immunofluorescence can be used to demonstrate the presence of von Willebrand factor (VWF), or other endothelial surface markers (Table 1).

Another means of validation is through gene expression profiling assessing whether or not expression of endothelial genes such as vascular endothelial cadherin (VE-cadherin), is present in the population of cells designated as CEC (Smirnov *et al*, 2006).

## AVAILABLE ASSAYS

### Manual immunomagnetic isolation

The most widely used method to isolate CEC, is by the use of magnetic beads coupled to a monoclonal antibody (mAb) targeting CD146, as first described by Dignat-George (George *et al*, 1992). After isolation, additional techniques, such as flow cytometry or microscopic analysis, are used to identify CEC based on morphological or immunophenotypical criteria. A major advantage of this technique is that it permits visual identification of CEC, which allows them to be discriminated from endothelial micro particles, anuclear cells, and cellular conglomerates. However, manual bead-based isolation has several shortcomings, especially with regard to large monitoring studies. It is labour-intensive, requires a high level of operator skills, and requires additional steps to positively identify CEC, which make them unsuitable for high throughput monitoring. Furthermore, magnetic enrichment may give rise to an underestimation of the actual number of CEC. As CD146 expression on CEC is lower than on the HUVEC used to test assay recovery, its expression by CEC might be insufficient to bind to magnetic beads.

### Automated isolation and staining

A variant on techniques applying a manual immunomagnetic enrichment step, is the CellTracks AutoPrep and CellTracks Analyzer II System (Immunicon Corp, Huntington Valley, PA, USA), initially designed to detect circulating tumour cells. As with manual immunomagnetic isolation, cells are isolated by CD146-coupled ferrofluids, with the main difference being that the CellTracks System is fully automated, and is therefore not operator dependent. After isolation, the suspension of CD146^+^ cells is stained with (i) 4′,6-diamidino-2-phenylindole (DAPI) to identify nucleated cells, (ii) CD105, fairly unique on endothelial cells, and (iii) CD45, to exclude CD146-expressing T cells. The enriched and stained sample is dispensed in a magnetic cartridge to form a monolayer of cells, which is scanned by the CellTracks Analyzer II System. The generated images are evaluated for CEC by visual inspection, in which CEC are defined as DAPI^+^, CD105^+^, CD146^+^, CD45^−^ (Rowand *et al*, 2007). The endothelial feature of the cells meeting these phenotypic criteria was further demonstrated by global gene expression profiling, which clearly demonstrated the presence of endothelial markers (Smirnov *et al*, 2006). Drawbacks of this method are the costly equipment and reagents, while assay customisation is not possible. Also, the maximum number of only eight samples that can be analysed in a single run, combined with the relative long duration of a complete run (approximately 4 h), limits high throughput analysis.

### Flow cytometry

Flow cytometry is a widely accepted tool for immunophenotyping of cells. A cocktail of fluorochrome-labelled mAb is used to identify CEC, allowing a highly specific definition of CEC, and possibly, the identification of endothelial cell subsets such as EPC. Another benefit of flow cytometry is, that by multi-colour staining cells with an immunophenotypical overlap with CEC, such as endothelial micro particles and platelets, can be excluded from evaluation, which increases specificity. However, flow cytometry assays in whole blood are at risk to overestimate CEC by enumerating false-positive cells. A well-known cause of false positivity is non-specific antibody-binding. Non-specific-binding can be the result of Fc receptor-binding, binding to dead cells, or by improper use of reagents. A recent study clearly showed that the exceptionally high CEC counts found with a commonly applied flow cytometry based assay (Mancuso *et al*, 2001), were due to the fact that the majority of the cells designated CEC were in fact large platelets (Strijbos *et al*, 2007).

As a result of improper titration of the CD146 mAb cells with a CD45^dim^, CD31^+^ phenotype stained positively for this antigen, suggesting that CEC were enumerated. Such problems can be prevented through staining whole blood rather than a cell suspension, or through the use of a blocking reagent, by which Fc receptors are kept saturated and non-specific binding is prevented. Furthermore, addition of a real-time viability stain, such as 7-aminoactinomycin D or propidium iodide, allows the exclusion of dead cells. Next to interference by non-specific antibody-binding, false positivity can also result from specific-binding to soluble forms of the antigen. Many endothelial surface antigens such as sCD144, and sCD146 are secreted, and their uptake by platelet aggregates can result in positivity. An effective strategy for excluding platelets, aggregates and endothelial micro particles from analysis is by using a DNA-specific stain, such as (DAPI) or 1,5-bis[2-(di-methylamino) ethyl]amino-4, 8-dihydroxyanthracene-9,10-dione (DRAQ5).

Recently, absolute CEC counts obtained by both magnetic isolation and flow cytometry assays were compared (Goon *et al*, 2006). The reasonable agreement in CEC counts between both methods, in combination with an established CEC phenotype (i.e. CD34^+^, CD146^+^, CD45^−^), is suggestive that actual CEC counts are being approached by this flow cytometry assay. However, isolation and validation as done on the magnetically isolated cells (Rowand *et al*, 2007), was not performed on the CEC as defined by Goon *et al* (2006).

## CLINICAL RESULTS

Although a large number of studies on CEC in cancer patients have been published, many of these studies rely on a flow cytometric approach, which defines CEC as being CD45^−^, CD31^+^, and CD146^+^. Although this is a generally accepted CEC immunophenotype, reports are currently not available in which the endothelial origin of cells enumerated by those assays has been demonstrated unambiguously (Duda *et al*, 2006; Mancuso *et al*, 2006). As a result, studies on CEC that are in our opinion reliable, are scarce: only three studies are currently available, all based on CD146 driven magnetic isolation of CEC (Beerepoot *et al*, 2004; Woywodt *et al*, 2006; Rowand *et al*, 2007). In the first study, CEC were enumerated by magnetic isolation, followed by visual analysis of CD31, CD309 and VWF expression, determined by immunofluorescence. CEC numbers in cancer patients (*n*=146, mean CEC 399±36 ml^−1^) were found to be increased 3.3 fold when compared to healthy controls (*n*=46, mean CEC 121±16 ml^−1^). Further analysis of CEC numbers in cancer patients, demonstrated significantly higher CEC in patients with progressive disease *vs* those with stable disease (95 patients, 438±65 CEC per ml and 17 patients, 179±61 CEC per ml, respectively), whereas no difference was seen between patients with stable disease and healthy donors (Beerepoot *et al*, 2004). The increased CEC count in cancer patients was confirmed in a study of Rowand using the CellTracks System. Cells were isolated by automated CD146 driven magnetic isolation, and assay accuracy, sensitivity, linearity and precision were assessed, Hereafter, nucleated (DAPI^+^) cells, expressing CD105 but lacking CD45 were enumerated in 249 healthy donors and 206 patients with metastatic cancer (Rowand *et al*, 2007). The observed mean CEC count of 21 in healthy controls is comparable by those found by the EUROCEC assay. Strikingly, the number of CEC reported in healthy donors was 10-fold lower when compared to those reported by Beerepoot, namely 0–20 ml^−1^ (Rowand *et al*, 2007). Both groups isolate CEC using beads targeting CD146, but Beerepoot uses CD31, CD309 and VWF, rather than CD105 to confirm the endothelial origin of cells. Where CD31 and VWF are considered to be pan-endothelial cell markers, CD105 is expressed predominantly on proliferative and/or malignant CEC. Given its high recovery and reproducibility and extensive validation, we favour the CellTracks assay for isolating CEC.

## POTENTIAL APPLICATIONS IN ONCOLOGY

Provided that an adequately validated, sensitive and specific assay is used, the detection and enumeration of CEC in patients with solid malignancies offer a wide spectrum of potential applications.

### CEC: marker for establishing prognosis and for follow-up?

The observation that advanced cancer patients have higher CEC counts than healthy controls (Beerepoot *et al*, 2004; Rowand *et al*, 2007), whereas patients with stable disease and healthy individuals have similar numbers (Beerepoot *et al*, 2004), may imply that CEC enumeration may be used for prognosis and during follow-up in cancer patients. Obviously, verification of this statement would require studies in which large numbers of patients are included with enumeration of CEC at baseline and during follow-up.

### CEC: marker for response to treatment?

Classic methods to establish antitumour effects of systemic agents, which rely on assessing changes in tumour size by radiological assessments, are no longer sufficient. This holds true not only when using conventional chemotherapeutic drugs, but in particular when angiogenesis inhibitors are applied. In several randomised trials exploring such drugs, enhanced progression-free periods were observed, yet the response rates as defined by RECIST, were minimal (Yang *et al*, 2003). Such findings emphasize the limitations of conventional imaging, as well as the need to incorporate novel, sensitive indicators of response. The increase in CEC numbers in mice stimulated with VEGF, and the consecutive decrease of these numbers after treatment with the angiogenesis inhibitor endostatin (Schuch *et al*, 2003), are suggestive of a possible role for CEC in monitoring response to treatment when using agents targeting the VEGF-VEGFR pathway. Recently, a xenograft prostate cancer model showed that an increase in apoptotic CEC after treatment with thalidomide and docetaxel, was predictive for response to treatment (Li *et al*, 2008). However, as these results were based on a non-validated assay, it is important to confirm these findings using different, validated techniques.

### CEC: guideline for optimal drug dosing?

Nowadays, it is increasingly recognised that the recommended dose for further exploration of a drug should be the optimal biological drug dose (OBD) rather than the MTD. The OBD is defined as the dose that is feasible to be applied in humans and yields biological effects. Changes in CEC counts after dose escalation might provide useful insights in establishing the OBD when assessing agents targeting vasculature such as VDA or angiogenesis inhibitors. A recent study by Celik *et al* (2005) reports a 50% decrease in CEC in tumour-bearing mice treated with endostatin. The decrease in CEC showed a clear U-shaped dose relation. The optimum dose, determined by assessment of tumour micro vessel density and analysis of tumour blood flow, resulted in the largest decrease in CEC numbers, whereas under- or over treatment resulted in a diminished decrease or even increase in CECs, suggesting a rationale for using CEC as guideline for optimum drug dosing. However, whether this holds true for humans, and for anti-angiogenic drugs other than endostatin, remains to be established.

### CEC: towards identification of new targets in oncology?

One of the major advances in the management of cancer in the last decades is the introduction of targeted therapy. By identifying tumour factors that play a crucial role in the pathogenesis of a disease, an avalanche of new targets for therapy has been identified. Examination of endothelial cells from tumour vasculature, may result in the identification of antigens specific for malignant angiogenesis, such as the recently identified H3 homologue of the costimulatory molecule B7 (CD276) (Seaman *et al*, 2007), and allow the development of agents selectively targeting tumour vasculature.

### CEC: marker for vascular toxicity?

With better treatment outcomes for some patient populations with advanced malignancies, there is growing interest for long-term side effects of antitumour therapies. An increased incidence of cardiovascular events has emerged as one of the most important long-term untoward sequelae. For example, patients treated with chemotherapy for advanced germ-cell cancer have a 2–7-fold increased risk for cardiovascular events when compared to the general population (Meinardi *et al*, 2000). Although the exact mechanism of this increased risk is unknown, *in vitro* data suggest that cytotoxic agents directly cause endothelial damage. Frequently used agents such as cisplatin, bleomycin and etoposide, can cause thickening of the carotid artery intima, Raynaud's phenomenon, and an increase in plasma C-reactive protein (CRP), VWF and PAI-1, all associated with endothelial dysfunction (Nuver *et al*, 2005). Monitoring biomarkers of endothelial dysfunction or damage such as CEC levels, both during and after treatment might provide more insight into the vascular toxicity profile of chemotherapeutic agents. On the basis of such data, less vasotoxic treatments with equivalent antitumour activity would be warranted, especially for patients for whom treatment is likely to result in long-term survival, such as in germ cell cancers, childhood lymphoblastic leukaemia and lymphoma subtypes. A recent phase I study with the protein kinase β C inhibitor enzastaurin (Rademaker-Lakhai *et al*, 2007), in which CEC were enumerated by immunomagnetic isolation, did not find an effect of enzastaurin on CEC numbers. In contrast, a study on the VDA ZD6126 demonstrated a significant increase in immunomagnetically isolated CEC 2–8 h after infusion (Beerepoot *et al*, 2004). Given the presumed mechanism of action of VDAs, this finding strongly suggests that CEC serves as a marker to assess vascular toxicity of drugs.

## CONCLUSIONS

In view of the growing recognised role of angiogenesis in oncology, and the integration of drugs targeting tumour vasculature, biomarkers that enable monitoring effects on vasculature are urgently needed. Many soluble markers, including VWF and thrombomodulin have been proposed as such markers. However, most of these are acute phase products and therefore susceptible to interference by other events frequently encountered in cancer patients such as infection. By contrast, monitoring CEC appears an attractive candidate. Several techniques have been developed to detect and enumerate CEC, but the lack of consensus on the phenotype of CEC, as well as their low numbers in blood, have resulted in conflicting results and have severely hindered progress in this important field. Providing a clear definition of CEC together with careful confirmation and validation that cell population designated ‘circulating endothelial cells’ are indeed CEC, are the most important issues that need to be addressed. To overcome these issues, suggestions on phenotype and detection strategy have been provided by the EUROCEC network (Woywodt *et al*, 2006). Despite these problems, validated assays enabling the proper enumeration of CEC are since recently available and the first results have revealed that patients with advanced cancer have indeed higher levels than healthy controls. In theory, there are many potential applications for the assessment of CEC counts in patients with solid malignancies including establishing prognosis and therapy-induced effects, but whether or not CEC can actually be used for these purposes is currently the subject of clinical studies. So only time will tell whether CEC can fulfil its promise as a biomarker in clinical oncology.

## Figures and Tables

**Table 1:** Markers used in CEC assays

**Marker**	**Description**	**Subtype association**	**Expression level**	**Coexpression**	**References**
CD31	PECAM-1	Pan-endothelial	++^a^	P, Pan-leukocyte	(Beerepoot *et al*, 2004)
CD34	Stem cell marker	Pan-endothelial	++^a^	S	(Furstenberger *et al*, 2006)
CD36	Collagen receptor I	Micro vascular	+^a^	P, E, M, D	(Moroni *et al*, 2005)
CD54	ICAM-1	Inflammation	+^a^	L, M	(Dixon *et al*, 2004)
CD62-E	E-selectin	Inflammation	++	—	(Dixon *et al*, 2004)
CD62-P	P-selectin	Inflammation	+	P	(Corcoran *et al*, 2006)
CD105	Endoglin	Angiogenesis, malignant	++^a^	S, M^b^	(Rowand *et al*, 2007)
CD106	VCAM-1	Inflammation, malignant	+^a^	—	(Dixon *et al*, 2004)
CD137	ILA/4	Malignant	+	L^b^, D	(Seaman *et al*, 2007)
CD144	VE-cadherin	Pan-endothelial	+^a^	—	(Smirnov *et al*, 2006)
CD146	MelCAM	Pan-endothelial	++^a^	L^b^	(Dignat-George *et al*, 2007)
CD202b	Tie-2	Angiogenesis	(+)^a^	—	(Smirnov *et al*, 2006)
CD276	B7-H3	Malignant	+	D, M^b^	(Seaman *et al*, 2007)
CD309	VEGFR-2	Angiogenesis	(+)^a^	S	(Beerepoot *et al*, 2004)
VWF		Pan-endothelial	++^a^	P	(Woywodt *et al*, 2006)
UEA-1		Pan-endothelial	++	—	(Woywodt *et al*, 2006)
DiL-AcLDL		Pan-endothelial	++	—	(Voyta *et al*, 1984)

PECAM-1=platelet/endothelial cell adhesion molecule 1, ICAM-1=intracellular adhesion molecule 1, VCAM-1=vascular cellular adhesion molecule 1, ILA/4=inducible by lymphocyte activation /4, MelCAM=melanoma-associated cellular adhesion molecule, Tie-2=angiopoietin-1, 2, 4 receptor, B7-H3=B7 homologue 3, VEGFR-2=vascular endothelial growth factor receptor 2. S=hematopoietic stem cells, L=lymphocytes, P=platelets, M=monocytes, E=erythrocytes, D=dendritic cells.

Expression levels: ++=strong, +=moderate, (+)=weak.

a Indicates data based on flow cytometric results from the authors.

b Indicates presence on activated cells.
